# Serum, liver and bile sitosterol and sitostanol in obese patients with and without NAFLD

**DOI:** 10.1042/BSR20171274

**Published:** 2018-04-20

**Authors:** Milla-Maria Tauriainen, Ville Männistö, Dorota Kaminska, Maija Vaittinen, Vesa Kärjä, Pirjo Käkelä, Sari Venesmaa, Helena Gylling, Jussi Pihlajamäki

**Affiliations:** 1 Department of Medicine, University of Eastern Finland and Kuopio University Hospital, Finland; 2 Institute of Public Health and Clinical Nutrition, University of Eastern Finland, Finland; 3 Department of Pathology, University of Eastern Finland and Kuopio University Hospital, Kuopio, Finland; 4 Department of Surgery, University of Eastern Finland and Kuopio University Hospital, Kuopio, Finland; 5 University of Helsinki and Helsinki University Central Hospital, Internal Medicine, Helsinki, Finland; 6 Clinical Nutrition and Obesity Center, Kuopio University Hospital, Kuopio, Finland

**Keywords:** biliary samples, gene expression, liver samples, nafld, plant sterols and stanols, sitostanol

## Abstract

**Background and aims**: Non-alcoholic fatty liver disease (NAFLD) associates with low levels of serum plant sterols in cross-sectional studies. In addition, it has been suggested that the hepatic sterol transport mechanisms are altered in NAFLD. Therefore, we investigated the association between serum, liver and bile plant sterols and sitostanol with NAFLD.

**Methods**: Out of the 138 individuals (age: 46.3 ± 8.9, body mass index: 43.3 ± 6.9 kg/m², 28% men and 72% women), 44 could be histologically categorized to have normal liver, and 94 to have NAFLD. Within the NAFLD group, 28 had simple steatosis and 27 had non-alcoholic steatohepatitis. Plant sterols and sitostanol were measured from serum (*n*=138), liver (*n*=38), and bile (*n*=41). The *mRNA* expression of genes regulating liver sterol metabolism and inflammation was measured (*n*=102).

**Results**: Liver and bile sitostanol ratios to cholesterol were higher in those with NAFLD compared to those with histologically normal liver (all *P*<0.022). Furthermore, liver sitostanol to cholesterol ratio correlated positively with histological steatosis and lobular inflammation (*r*_s_ > 0.407, *P*<0.01 for both). In contrast, liver sitosterol to cholesterol ratio correlated negatively with steatosis (*r*_s_ = −0.392, *P*=0.015) and lobular inflammation (*r*_s_ = −0.395, *P*=0.014). Transcriptomics analysis revealed suggestive correlations between serum plant sterol levels and mRNA expression.

**Conclusion**: Our study showed that liver and bile sitostanol ratios to cholesterol associated positively and liver sitosterol ratio to cholesterol associated negatively with liver steatosis and inflammation in obese individuals with NAFLD..

## Introduction

Nonalcoholic fatty liver disease (NAFLD) is the most common cause of liver injury in Western countries [1]. NAFLD can present as simple steatosis, but it can also proceed into nonalcoholic steatohepatitis (NASH), and ultimately to liver fibrosis and cirrhosis [2]. Currently, the mechanisms regulating the progression from steatosis to NASH are poorly defined.

NAFLD associates with low levels of serum plant sterols in cross-sectional studies [3,4] and plant sterols are suggested to prevent the progression of NAFLD [5]. Plant sterols and plant stanols are normal components of plants. They cannot be synthesized in humans and are therefore completely derived from food. The most frequent plant sterols present in humans are campesterol, sitosterol and avenasterol, and the most frequent plant stanol is sitostanol [6]. Thus, the serum levels of plant sterols, especially as ratios to serum cholesterol concentration, are used as biomarkers of cholesterol absorption efficiency [7–9]. Accordingly, their low serum levels reflect decreased intestinal absorption of sterols, e.g. in insulin resistant states [10] including NAFLD and NASH [3,4].

Absorption of sterols from the small intestine and biliary excretion from the liver and bile are regulated by transporter genes Niemann–Pick C1-Like 1 (*NPC1L1*), ATP-Binding Cassette, Subfamily G, Member 5 (*ABCG5*), and ATP-Binding Cassette, Subfamily G, Member 8 (*ABCG8*) [11,12]*.* For example, *ABCG5/8* deficiency reduces cholesterol excretion from the liver into the bile [13–15] and increases cholesterol absorption in mice [15] and in humans [14]. On the other hand, normally functioning NPC1L1 transporter located at the hepatic canalicular membranes actively transports sterols into hepatocytes [16]. Interestingly, liver protein expression of ABCG8 and ABCG5 has been suggested to be higher and expression of NPC1L1 to be lower in those with steatosis and NASH compared to those with normal liver [17,18]. On the other hand, both the mRNA and protein expression of ABCG8 has been reported to be lower in those with NAFLD or NASH than in those with normal liver [19]. Taken together, these results suggest a link between altered sterol/stanol export mechanisms and NAFLD.

To clarify the mechanisms for altered plant sterol and plant stanol metabolism in NAFLD and NASH, we investigated serum, liver and biliary plant sterol (campesterol, sitosterol, and avenasterol) and sitostanol levels in 138 obese individuals participating in the Kuopio Obesity Surgery Study (KOBS).

## Materials and methods

### Subjects

All patients undergoing obesity surgery in Kuopio University Hospital are recruited into our ongoing study investigating the metabolic consequences of obesity surgery (Kuopio Obesity Study, KOBS) [20,21].

**Figure 1:**
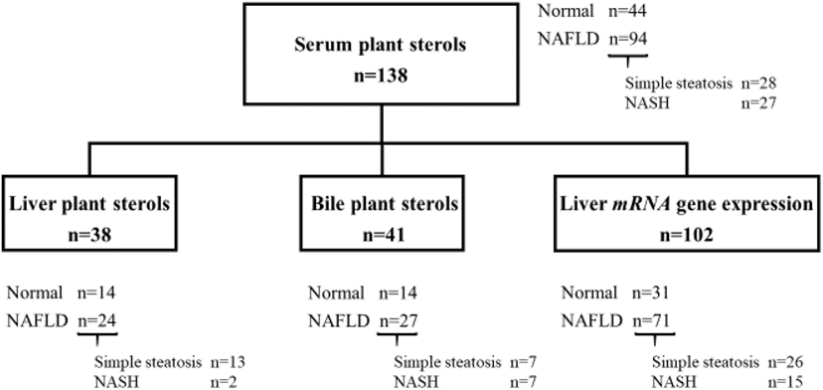
A chart demonstrating the study subjects in groups that had serum, liver and bile measurements of plant sterols and liver mRNA expression available Of the original cohort of 150 subjects that had serum plant sterol measurements available, a distinct liver phenotype could be recognized in 138 subjects [normal liver (normal, *n*=44) and nonalcoholic fatty liver disease (NAFLD, *n*=94)]. Of those in NAFLD group, 28 had simple steatosis and 27 had nonalcoholic steatohepatitis (NASH). Of the 138 subjects who had serum plant sterol measurements, liver (*n*=38) and bile (*n*=41) sterol measurements, and liver mRNA expression (*n*=102) were performed. Liver and bile sterol measurements were from different subjects.

The study group included 138 individuals from the KOBS [mean age: 46.3 ± 8.9, body mass index (BMI): 43.3 ± 6.9 kg/m², 38 (28%) men and 100 (72%) women], of whom the measurements of serum plant sterols were available and the histological liver phenotype was either normal or NAFLD. Subjects using cholesterol lowering medications were excluded. Forty-four of the 138 participants had histologically normal liver and 94 had NAFLD. From those who had NAFLD, 28 had simple steatosis and 27 had NASH, and the remaining 39 participants with NAFLD had an intermediate phenotype between simple steatosis and NASH and were thus excluded from the study groups with specified phenotypes (Figure 1). Plant sterols and sitostanol were measured from serum (*n*=138), liver (*n*=38), and bile (*n*=41). The mRNA expression of genes *NPC1L1, ABCG5* and *ABCG8*, and several other genes regulating inflammation and lipid metabolism in the liver, was measured from liver samples of 102 individuals (Figure 1).

The study protocol confirms to the ethical guidelines of the 1975 Declaration of Helsinki (6th revision, 2008) as reflected in a prior approval by the institution’s human research committee, and has been approved by the Ethics Committee of the Northern Savo Hospital District (54/2005, 104/2008, and 27/2010). Written informed consent was obtained from each patient included in the study.

### Laboratory measurements

Cholesterol and triglycerides from serum were assayed by an automated enzymatic method (Roche Diagnostics, Mannheim, Germany), as described before [10,21]. Plant sterols (campesterol, sitosterol, and avenasterol) and sitostanol were measured in serum (*n*=138), liver (*n*=38), and bile (*n*=41) by gas–liquid chromatography (GLC) with a 50 m long capillary column (Ultra 2; Agilent Technologies, Wilmington, DE) as described earlier [21] with 5α-cholestane as the internal standard. To standardize the varying cholesterol levels, the plant sterols and sitostanol concentrations in serum, liver, and bile are presented as ratios to cholesterol by dividing the plant sterol and sitostanol concentrations with the respective cholesterol concentration of the same GLC run. Dietary phytosterol intake (DPI) was considered by calculating the ratio serum campesterol/cholestanol [22]. The serum plant sterol and sitostanol values are expressed as 10² mmol/mol cholesterol (the multiplication with 10² was used to reduce the decimals), those of liver as µg/100 mg of liver cholesterol, and those of bile as µg/100 mg of cholesterol respectively.

### Liver biopsies, bile samples and histological study groups

Liver biopsies were obtained using Trucut needle (Radiplast AB, Uppsala, Sweden) or with the ultrasonic scissors during elective laparoscopic Roux-en-Y gastric bypass (RYGB) operation. Overall the histological assessment of liver biopsy samples was performed by one pathologist according the standard criteria [23,24]. According to histology, patients were divided into two main study groups: normal liver (no steatosis, inflammation, ballooning, or fibrosis) and NAFLD (>5% of the hepatocytes have lipid droplets). From those who had NAFLD, a subdivision was possible for simple steatosis (>5% steatosis without inflammation, ballooning, or fibrosis) and NASH, as previously described [25]. Thirty-nine subjects could not be categorized to specified phenotypes with simple steatosis and NASH (Figure 1). However, all study subjects were included in correlation analyses (Table 2). Bile sample was taken transhepatically from the gall bladder during the operation with a fine needle aspiration.

### Liver gene expression

All samples for gene expression analysis were immediately frozen in liquid nitrogen. Total RNA from the liver tissue was extracted using Tri-Reagent (Applied Biosystems [ABI] Foster City, CA) and reverse-transcribed using the High Capacity cDNA Reverse Transcriptional KIT (ABI) according the manufacturer’s protocol. Quantitative real-time polymerase chain reaction (PCR) was carried out with the Applied Biosystems 7500 Real Time PCR System using KAPA SYBR FAST qPCR Universal Master Mix (KAPA Biosystems, Woburn, MA). Primers are listed in Supplementary Table S1. Relative expression was normalized to *RPLP0*. A gene panel of TruSeq Targeted RNA Expression (TREx) platform with MiSeq system (Illumina, San Diego, CA, U.S.A.) was also used for measuring gene expression levels in the liver at baseline of the KOBS study, as previously described [25].

For the TREx analysis, total RNA from the liver (150 ng) was reverse-transcribed using the ProtoScript II Reverse Transcriptase (New England BioLabs). The oligo pool targeted regions of interest were hybridized to cDNA. Next, hybridized cDNA was extended by DNA polymerase followed by ligation using DNA ligase. The extension–ligation products were amplified with PCR and AMPure XP beads (Beckman Coulter) were used to clean up the PCR products. Equal volumes of the products were pooled together and quantitated with DNA 1000 chip (Agilent Technologies). Finally, the pooled sample was diluted, denatured, and sequenced with MiSeq.

### Statistical analysis

All analyses were conducted via IBM SPSS Statistics for Windows, Version 21, (Armonk, NY: IBM Corp). Data are presented as mean ± standard deviation (SD). Differences between the study groups were examined by the χ2 (in categorical variables) and by nonparametric Kruskal–Wallis test (continuous variables). The Spearman rank correlation was used for correlation analysis. For the TREx analysis, the expression levels for each gene per sample in the gene panel were normalized based on the total number of aligned reads of the corresponding sample.

## Results

### Clinical characteristics

Table 1 demonstrates characteristics of the 138 participants (38 men and 100 women) in the study groups with normal liver and NAFLD. Age and BMI did not differ between the groups. Serum alanine aminotransferase (ALT) (*P*=0.007), fasting plasma glucose, and insulin levels were higher in those with NAFLD compared to those with normal liver (*P*<0.001). DPI was not different between the study groups (Table 1). The characteristics of study subjects in subgroups that had plant sterol and plant stanol measurements available from liver (*n*=38) and bile (*n*=41) are shown in Supplementary Table S2.

**Table 1:** Clinical characteristics (mean ± SD) of study subjects divided to those with normal liver and nonalcoholic fatty liver disease (NAFLD)

	Normal liver	NAFLD	*P* over the groups
	44	94	
Gender (male/female)	12/32	26/68	0.962
Age (years)	44.2 ± 8.4	47.2 ± 9.0	0.069
Body mass index (kg/m²)	43.5 ± 5.7	43.3 ± 7.4	0.911
ALT (U/L)	39.7 ± 28.3	54.3 ± 34.7	**0.007**
Fasting glucose (mmol/l)	5.7 ± 0.8	6.8± 2.3	**0.001**
Fasting insulin (mU/l)	14.2 ± 7.3	22.0 ± 11.9	**0.0004**
Total cholesterol (mmol/l)	4.4 ± 0.7	4.5 ± 1.0	0.831
HDL cholesterol (mmol/l)	1.1 ± 0.3	1.1 ± 0.3	0.987
LDL cholesterol (mmol/l)	2.7 ± 0.6	2.7± 0.9	0.805
Total triglycerides (mmol/l)	1.5 ± 0.6	1.6 ± 0.7	0.204
DPI*(dietary phytosterol intake)	0.96 ± 0.4	0.98 ± 0.4	0.971

*P*<0.05 compared with normal liver; *DPI (dietary phytosterol intake, serum campesterol to cholestanol ratio).

### Serum plant sterols and sitostanol do not associate with liver histology

Serum plant sterols and sitostanol ratios to cholesterol did not differ between the study groups (Supplementary Figure S1). Accordingly, serum levels of plant sterols and sitostanol did not correlate with histological parameters (Table 2).

**Table 2:** Spearman correlations of serum and liver plants sterols and sitostanol (ratio to total cholesterol) with liver histology

	Steatosis grade	Fibrosis stage	Lobular inflammation	Ballooning
**Serum (*n*=138)**				
Campesterol	−0.025	−0.002	−0.025	0.092
Sitosterol	−0.027	0.029	−0.028	0.159
Avenasterol	0.092	0.099	0.086	0.128
Sitostanol	0.100	0.026	0.098	0.024
**Liver (*n*=38)**				
Campesterol	0.013	0.137	−0.052	0.119
Sitosterol	−0.392*	−0.097	−0.395*	0.054
Avenasterol	0.041	0.086	−0.025	−0.107
Sitostanol	**0.650^†^**	0.215	0.407*	0.059

Significant correlations are bolded, *P<0.05, ^†^*P*<0.01.

### Liver sitosterol and sitostanol ratios to cholesterol associate with liver steatosis and inflammation

**Figure 2:**
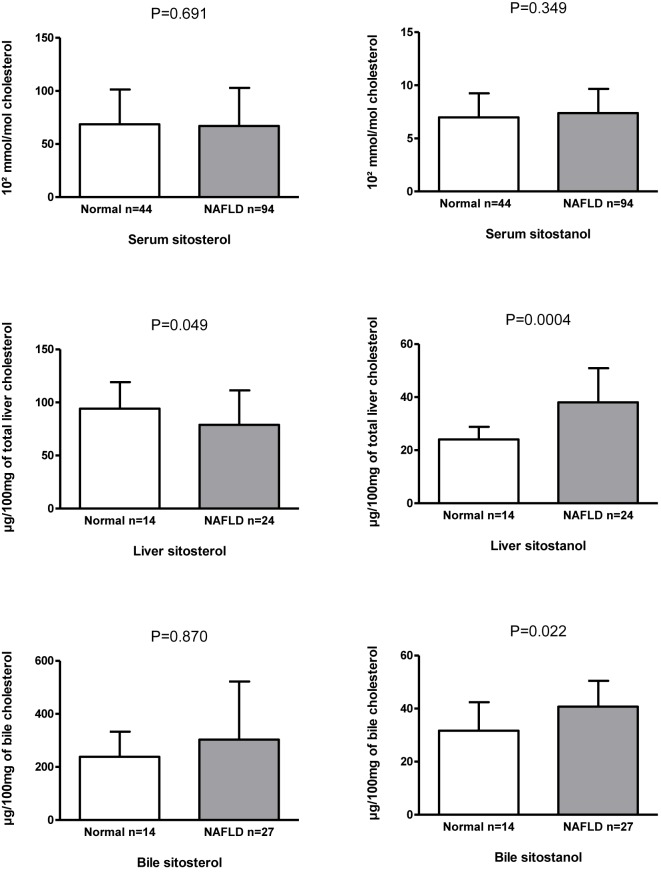
Serum, liver and bile sitosterol and sitostanol ratios to cholesterol (mean ± SD) in individuals with normal liver and nonalcoholic fatty liver disease (NAFLD).

Liver sitosterol ratio to cholesterol was lower and that of liver sitostanol was higher in those subjects with NAFLD compared to individuals with normal liver (*P*=0.049 and *P*=0.004) (Figure 2). Accordingly, liver sitosterol ratio to cholesterol correlated inversely with steatosis and lobular inflammation (*r*_s_ < −0.392, *P*<0.015 for both), whereas liver sitostanol ratio to cholesterol correlated positively with liver steatosis and inflammation (*r*_s_ > 0.407, *P* < 0.011 for both) (Table 2). Liver avenasterol and campesterol ratios to cholesterol did not associate with NAFLD (data not shown), nor did they correlate with steatosis or inflammation (Table 2). Liver and serum campesterol, sitosterol, and avenasterol ratios to cholesterol correlated with each other (*n*=38, *r*_s_ = 0.544–0.488, *P*<0.02 for all), but liver and serum sitostanol ratios to cholesterol did not correlate with each other (Supplementary Table S3).

### Biliary sitostanol ratio to cholesterol is increased in individuals with steatosis

Finally, we measured plant sterols and sitostanol from the bile (*n*=41). Sitostanol ratio to cholesterol was higher in those with NAFLD than those with normal liver (*P*=0.022, Figure 2) while biliary sitosterol ratio to cholesterol did not differ between the study groups (Figure 2). In addition, there was a strong positive correlation between serum and biliary sitosterol (*r*_s_ = 0.795, *P*=1.45 × 10^−9^), but not between serum and bile sitostanol ratios to cholesterol (Supplementary Table S3). Campesterol and avenasterol were unmeasurable in the biliary samples.

### Liver mRNA expression with plant sterols and liver histology

**Figure 3:**
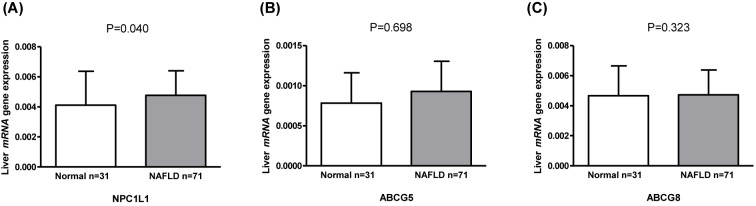
Liver mRNA expression analyzed with qPCR (mean ± SD) of *NPC1L1* (Niemann–Pick C1-Like 1) (**A**), *ABCG5* (ATP-Binding Cassette, Subfamily G, Member 5) (**B**), and *ABCG8* (ATP-Binding Cassette, Subfamily G, Member 8) (**C**) in individuals with normal liver and nonalcoholic fatty liver disease (NAFLD).

Next, we investigated if the differences in sitostanol and sitosterol levels could be related to the liver mRNA expression of transporters *NPC1L1, ABCG5*, and *ABCG8* (*n*=102)*.* First, we observed that the hepatic mRNA expressions of *NPC1L1* was higher in those with NAFLD compared to those with normal liver (*P*=0.040) (Figure 3A). *ABCG5* and *ABCG8* were not different between the study groups (Figure 3B,C). Next, we correlated the liver *mRNA* expression of these genes with sitosterol and sitostanol ratios to cholesterol in serum (*n*=102), liver (*n*=38), and bile (*n*=41) (Supplementary Tables S4 and S5). The mRNA expression of *NPC1L1* correlated negatively with serum sitosterol (*r*_s_ = −0.210, *P*=0.032) and positively with serum sitostanol (*r*_s_ = 0.248, *P*=0.011), but not with liver or bile sitosterol or sitostanol ratios to cholesterol. Finally, we did a correlation analysis between the expression of several other known genes regulating inflammation, cholesterol and lipid metabolism, and the ratios to cholesterol of serum, liver and bile sitosterol and sitostanol (Supplementary Table S5). This analysis revealed several suggestive differences in correlations between mRNA expression and the sitostanol and sitosterol levels. However, due to the multiple testing of correlations none of the correlations were strongly significant and thus require further replication.

## Discussion

Our main finding was that liver sitosterol and sitostanol ratios to cholesterol associated differentially with normal liver and NAFLD in obese individuals (Figure 2). In contrast, we did not observe an association between liver histology and the levels of plant sterols and sitostanol in serum (Table 2). This suggests that serum sitosterol and campesterol ratios to cholesterol, are not primarily affected in NAFLD. More likely, a differential regulation of sitosterol and sitostanol contents in the liver may exist between those with normal liver and NAFLD.

There are several potential explanations why liver sitosterol and sitostanol were differentially associated with NAFLD in our study. Even though serum and liver plant sterols correlated with each other, serum and liver sitostanol did not correlate suggesting different regulation of sitostanol (Supplementary Table S3). In addition, there was a strong positive correlation between serum and biliary sitosterol, but not between serum and biliary sitostanol suggesting that serum sitostanol levels do not reflect hepatic and biliary levels of sitostanol (Supplementary Table S3). First, this might be due to different chemical structures of sitosterol and sitostanol, which affect the solubility regulating their absorption and secretion [26–29]. Second, the positive correlation of liver sitostanol and negative correlation of liver sitosterol with liver inflammation (Table 2) suggest that their abilities to take part in inflammatory processes may differ. It is not yet clear how plant sterols and plant stanols can regulate inflammation in humans [30–32]. Plant sterols and stanols have been suggested to reduce inflammation in asthma both *in vitro* [33,34] and in animal models [33,35]. In addition, sitosterol and sitostanol markedly decreased the mRNA levels of *MCP-1* and *IL-1β* in cultured myofibroblasts from stenotic hearth valves [32]. Plant sterols and plant stanols have been reported to attenuate inflammatory responses via T-lymphocytes in cell models [34,36] and in humans [37], and via cytokines in animal and *in vitro* studies [33,36].

The finding of the association between NASH-related histological parameters and sitostanol was supported by remarkable positive correlations of liver sitostanol with steatosis and lobular inflammation. At the same time serum sitostanol levels did not correlate with histology (Table 2). Besides liver sitostanol biliary sitostanol levels are also positively associated with NAFLD (Figure 2). This is in line with experimental models in rats demonstrating that perfused sitostanol was taken into the isolated liver and secreted to bile [33,36]. Thus, our observation using bile samples in the analysis strengthens the conclusion that liver sitostanol metabolism is altered in NAFLD. Taken together, these results suggest that transport of sitosterol and sitostanol from gut to serum and further from the liver to bile may be differentially regulated in NAFLD compared with normal liver.

Our results of the liver mRNA expression of known genes involved in cholesterol, lipid, and inflammation metabolism suggest differences in sterol export mechanisms in NAFLD. Previously, the expression findings related to sterol exporters have been controversial in humans with NAFLD. ABCG5/8 protein expression was reported to be higher in those with steatosis compared to those with normal liver [17], a finding not confirmed in our study. In another study, the mRNA expression of *ABCG8* was found to be lower in humans with NASH compared to those with NAFLD while no difference in the expression of *ABCG5* was observed [19]. On the other hand, *NPC1L1* expression has been reported to be lower in those with NAFLD compared to those with normal liver [17]. This was opposite to our findings demonstrating that the liver mRNA expression of *NPC1L1* was higher in those with NAFLD compared to those with normal liver, whereas *ABCG5* and *ABCG8* were not changed (Figure 3A–C). Accordingly, serum sitosterol correlated negatively and sitostanol positively with the liver gene expression of *NPC1L1* (Supplementary Table S4), suggesting a link between our results and *NPC1L1* expression in the liver. However, our key finding that liver/bile sitostanol ratio to cholesterol was higher in those with NAFLD could not be linked to mRNA expression of export genes, supporting the possibility of a more complex dysregulation in NAFLD.

Our large-scale analysis of mRNA expression using Truseq methodology suggested other potential divergent metabolism between human serum, liver and bile metabolism of plant sterols and plant stanols. We saw differential correlations of sitosterol and sitostanol with the liver mRNA expression of known genes involved in inflammation, cholesterol, and lipid metabolism (Supplementary Table S5).

We recognize the following limitations in our study. Our study subjects were morbidly obese and thus our results cannot be generalized to normal weight subjects. However, it would be ethically challenging to obtain liver biopsies and bile samples from lean and healthy individuals. Unfortunately, we only had two individuals with NASH, as compared to 13 with simple steatosis, with liver samples available for liver analysis of plant sterols and sitostanol. Thus, we could not investigate the independent associations of liver sitostanol with steatosis and NASH.

In conclusion, our study is the first to demonstrate that both liver and bile sitostanol ratio to cholesterol associate with NAFLD, even though serum sitostanol ratio to cholesterol does not in obese individuals. The mechanisms related to altered sitostanol metabolism in NAFLD should be clarified in experimental studies.

## Clinical perspectives

- Association between plant sterols, sitostanol, and NAFLD is not clear. Thus, we studied serum, liver, and bile plant sterols in obese individuals with and without NAFLD.
- The main findings were that liver and bile, but not serum, sitostanol was higher in those with NAFLD compared to those with normal liver. Accordingly, liver sitostanol correlated positively with steatosis and lobular inflammation.
- The mechanisms related to altered sitostanol metabolism in NAFLD should be clarified in experimental studies.

## Supporting information

**Supplementary Figure. 1:** Serum plant sterols and sitostanol ratios to cholesterol (mean ± SD) in individuals with normal liver (n=44) and NAFLD (n=94). Serum campesterol, sitosterol, avenasterol or sitostanol were not different between normal liver and NAFLD.

**Supplementary Table 1:** Sequences of qPCR primers.

**Supplementary Table 2:** Clinical characteristics (mean±SD) of study subjects in groups that had plant sterol and plant stanol measurement available from serum, liver and bile and had either histologically normal liver or NAFLD. *P<0.05 compared to those with serum measurements. DPI (dietary phytosterol intake, serum campesterol to cholestanol ratio).

**Supplementary Table 3:** Spearman correlations of serum, liver and bile plant sterol and sitostanol. **P<0.01 and *P<0.05. Bile avenasterol and campesterol were undetected.

**Supplementary Table 4:** Spearman correlations of liver mRNA gene expression (qPCR assay) with plant sterols and sitostanol ratios to cholesterol in serum, liver and bile. *P<0.05.

**Supplementary Table 5:** Correlations of the inflammation, lipid and cholesterol metabolism associated genes (liver *mRNA* expression, qPCR assay) with sitosterol and sitostanol ratios to cholesterol in serum (n=102), liver (n=38) and bile (n=41). *P<0.05, **P<0.009 in bold. Spearman's correlation analysis. N/A: data not available.

## Abbreviations

NAFLD: Non-Alcoholic Fatty Liver Disease
NASH: Non-Alcoholic Steatohepatitis
KOBS: Kuopio Obesity Surgery Study
NPC1L1: Niemann-Pick C1-Like 1
ABCG5: ATP-Binding Cassette, Subfamily G, Member 5
ABCG8: ATP- Binding Cassette, Subfamily G, Member 8
RYGB: Roux-en-Y Gastric Bypass
ALT: Alanine aminotransferase
DPI: Dietary phytosterol intake
