# Supplementary material for: Serum, liver and bile sitosterol and sitostanol in obese patients with and without NAFLD
Source: Biosci Rep. 2018 Apr 20;38(2):BSR20171274. doi: 10.1042/BSR20171274 (PMC5968183; doi:10.1042/BSR20171274)

Serum, liver and bile sitosterol and sitostanol in obese patients with and without NAFLD

Tauriainen Milla-Maria, Männistö Ville, Kaminska Dorota, Maija Vaittinen, Kärjä Vesa, Käkelä Pirjo, Venesmaa Sari, Gylling Helena, Pihlajamäki Jussi

Table of contents

Supplementary Table 1 ……………………………………………………………………2

Supplementary Table 2 ……………………………………………………………………3

Supplementary Table 3 ……………………………………………………………………4

Supplementary Table 4 …………………………………………………………………...5

Supplementary Table 5 ……………………………………………………………………6-8

Supplementary Fig 1………………………………………………………………………9

Supplementary Table 1. Sequences of qPCR primers.

Gene	Forward primer (5' – 3')	Reverse primer (5' – 3')
NPC1L1	TACTGTGCCAATGCCCCGCT	GGGAAGACAGGGGCCCCGTA
ABCG5	TCTCATCTTTGACCCCCGGA	GATGTGATGTCCCACCAGGG
ABCG8	GGAACCTGGAGGGAACAATAAC	GGCATCTTGCTGGTACATCTT
RPLP0	GGCGACCTGGAAGTCCAACT	CCATCAGCACCACAGCCTTC

Supplementary Table 2. Clinical characteristics (mean±SD) of study subjects in groups that had plant sterol and plant stanol measurement available from serum, liver and bile and had either histologically normal liver or NAFLD. *P<0.05 compared to those with serum measurements. DPI (dietary phytosterol intake, serum campesterol to cholestanol ratio).

	**Serum**	**Liver**	**Bile**	**P serum vs. liver**	**P serum vs. bile**
	**138**	**38**	**39**		
Gender (male/female)	38/100	14/24	9/30	0.133	0.463
Age (years)	46.3±8.9	45.8±9.1	48.2±9.0	0.746	0.180
Body mass index (kg/m²)	43.3±6.9	43.8±5.2	41.6±4.9	0.662	**0.021**
ALT (U/L)	49.6±33.4	49.9±42.1	48.4±30.0	0.629	0.903
Fasting glucose (mmol/L)	6.5±1.9	6.3±1.1	6.4±1.7	0.604	0.932
Fasting insulin (mU/L)	18.6±10.1	18.5±10.5	18.8±9.5	0.694	0.673
Total cholesterol (mmol/L)	4.5±0.9	4.3±1.0	4.5±0.7	**0.031**	0.301
HDL cholesterol (mmol/L)	1.1±0.3	1.1±0.3	1.2±0.3	0.683	**0.004**
LDL cholesterol (mmol/L)	2.7±0.8	2.5±0.8	2.6±0.7	0.085	0.849
Triglycerides (mmol/L)	1.3±0.5	1.3±0.5	1.7±0.9	0.166	0.898
DPI (dietary phytosterol intake)	1.0±0.4	0.9±0.4	1.0±0.5	0.577	0.526

Supplementary Table 3. Spearman correlations of serum, liver and bile plant sterol and sitostanol. **P<0.01 and *P<0.05. Bile avenasterol and campesterol were undetected.

	Serum			
	Campesterol	Sitosterol	Avenasterol	Sitostanol
**Liver (n=38)**	**0.544****	**0.488****	**0.517***	0.214
**Bile (n=41)**		**0.795****		0.236

Supplementary Table 4. Spearman correlations of liver mRNA gene expression (qPCR assay) with plant sterols and sitostanol ratios to cholesterol in serum, liver and bile. *P<0.05.

.

	NPC1L1	***ABCG5***	***ABCG8***
**SERUM (n=102)**			
Campesterol	-0.112	-0.042	-0.090
Sitosterol	**-0.210***	-0.069	-0.172
Avenasterol	-0.046	0.117	-0.110
Sitostanol	**0.248***	**0.243***	-0.016
**LIVER (n=38)**			
Campesterol	-0.059	0.027	-0.194
Sitosterol	-0.168	-0.073	-0.310
Avenasterol	0.133	-0.025	**-0.383***
Sitostanol	0.215	0.190	0.012
**BILE (n=41)**			
Sitosterol	-0.295	-0.138	-0.209
Sitostanol	0.227	0.247	0.376

Supplementary Table 5. Correlations of the inflammation, lipid and cholesterol metabolism associated genes (liver *mRNA* expression, qPCR assay) with sitosterol and sitostanol ratios to cholesterol in serum (n=102), liver (n=38) and bile (n=41). *P<0.05, **P<0.009 in bold. Spearman`s correlation analysis. N/A: data not available.

Inflammation-related genes		Serum n=102		Liver n=38		Bile=41	
		Sitosterol	Sitostanol	Sitosterol	Sitostanol	Sitosterol	Sitostanol
Gene name	Gene symbol	10**2 mmol/mol of chol		ug/100 mg chol		ug/100mg chol	
ADAM Metallopeptidase Domain 8	*ADAM8*	-0,036	-0,069	-0,281	0,309	0,088	-0,211
Apoptotic Peptidase Activating Factor 1	*APAF1*	**,199***	0,054	0,174	**-,394***	0,227	-0,008
BCL2-Associated Agonist of Cell Death	*BAD*	-0,187	**-,205***	-0,088	0,065	**-,368***	0,050
BCL2-Associated Athanogene 6	*BAG6*	0,008	**-,386****	-0,262	0,064	-0,146	-0,200
B-Cell CLL/Lymphoma 3	*BCL2*	0,034	0,070	-0,185	0,243	0,067	0,178
Complement Component 3	*C3*	-0,105	**-,348****	-0,299	-0,013	-0,069	-0,107
Caspase 1, Apoptosis-Related Cysteine Peptidase	*CASP1*	-0,008	0,036	-0,255	0,203	-0,020	-0,077
Caspase 3, Apoptosis-Related Cysteine Peptidase	*CASP3*	-0,116	-0,001	0,043	0,043	-0,096	0,020
Caspase 8, Apoptosis-Related Cysteine Peptidase	*CASP8*	0,080	-0,165	-0,114	-0,091	0,013	-0,139
Caspase 9, Apoptosis-Related Cysteine Peptidase	*CASP9*	-0,013	**-,306****	-0,323	0,044	0,277	-0,102
Chemokine (C-C Motif) Ligand 2	*CCL2*	0,130	0,142	0,202	-0,133	0,079	-0,173
CD68 Molecule	*CD68*	-0,040	-0,039	-0,275	0,120	-0,147	0,273
C-Reactive Protein, Pentraxin-Related	*CRP*	-0,092	-0,020	-0,188	0,206	-0,022	-0,014
Egf-Like Module Containing, Mucin-Like, Hormone Receptor-Like 1	*EMR1=ADGRE1*	0,041	-0,024	0,085	0,105	-0,054	0,060
Fas (TNFRSF6)-Associated Via Death Domain	*FADD*	0,153	-0,017	0,042	-0,001	0,130	-0,170
Fas Cell Surface Death Receptor	*FAS*	**,238***	-0,030	0,064	-0,011	-0,036	-0,249
Guanylate Binding Protein 5	*GBP5*	-0,151	-0,009	-0,163	-0,117	-0,276	0,049
Heme Oxygenase 1	*HMOX1*	0,025	0,008	-0,024	0,282	-0,139	**,359***
Interleukin 10	*IL10*	-0,032	0,167	-0,119	0,266	0,310	0,294
Interleukin 18	*IL18*	0,041	-0,146	-0,144	0,116	-0,065	0,264
Interleukin 1, Beta	*IL1B*	-0,106	-0,068	-0,217	**,389***	-0,027	-0,162
Interleukin 1 Receptor, Type I	*IL1R1*	0,034	-0,125	-0,147	-0,172	0,151	0,016
Interleukin 1, Receptor, Type II	*IL1RN*	-0,175	0,066	-0,332	0,153	0,028	0,029
Interleukin 2	*IL2*	-0,040	0,067	-0,043	-0,216	0,025	0,041
Interleukin 32	*IL32*	-0,116	0,077	-0,281	**,528****	-0,062	0,003
Interleukin 6	*IL6*	-0,015	0,007	N/A	N/A	0,047	0,219
Integrin, Alpha M (Complement Component 3 Receptor 3 Subunit)	*ITGAM*	-0,018	-0,125	**-,437****	**,507****	0,120	-0,040
MHC Class I Pylypeptide-Related Sequence A	*MICA*	0,069	**-,240***	-0,158	-0,013	-0,120	**-,321***
Matrix Metallopeptidase 9	*MMP9*	-0,020	-0,010	-0,088	0,163	0,116	0,191
Nuclear Factor of Kappa Light Polypeptidase Gene Enhancer in B-Cells 1	*NFKB1*	-0,019	0,104	-0,016	0,239	-0,168	-0,235
nlr Family, Pyrin Domain Containing 3	*NLRP3*	-0,144	0,077	0,111	-0,129	-0,180	-0,055
Platelet Derived Growth Factor Beta Polypeptidase	*PDGFB*	-0,052	**-,194***	-0,289	0,099	**-,321***	0,180
PYD and Card Domain Containing	*PYCARD*	-0,023	0,023	0,104	-0,031	-0,116	0,236
Receptor (TNFRSF)-Interacting Serine-Threnonine Kinase 1	*RIPK1*	**,193***	0,190	0,228	-0,232	0,096	-0,059
Superoxide Dismutase 2, Mitochondrial	*SOD2*	-0,023	**,210***	**,361***	-0,119	-0,148	0,056
Signal Transducer and Activator of Transcription 5B	*STAT5B*	-0,007	0,042	-0,109	0,208	0,131	0,113
Toll-Like Receptor	*TLR4*	0,007	-0,143	**-,405***	**,391***	-0,035	-0,023
Tumor Necrosis Factor Alpha	*TNFα*	-0,060	0,038	-0,158	0,191	-0,172	0,051
Tumor Necrosis Factor Receptor Superfamily, Member 1A	*TNFRSF1A*	0,069	**-,213***	-0,123	-0,333	-0,037	-0,162
Tumor Necrosis Factor Receptor Superfamily, Member 1B	*TNFRSF1B*	-0,098	0,063	-0,310	0,112	-0,101	0,185
Tumor Necrosis Factor Receptor Superfamily, Member 10	*TNFSF10*	-0,008	**-,372****	-0,159	-0,290	0,112	-0,076
Tumor Protein 53	*TP53*	-0,032	-0,032	-0,215	0,160	0,164	-0,050
TNFRSF1A-Associated Via Death Domain	*TRADD*	-0,093	**-,222***	0,025	0,055	-0,086	-0,002
TNF Receptor-Associated Factor 2	*TRAF2*	-0,120	-0,088	-0,278	0,012	-0,305	0,027
Thioredoxin Interacting Protein	*TXNIP*	0,017	**,285****	0,193	0,126	0,070	0,245

Lipid metabolism - related genes		Serum n=102		Liver n=38		Bile=41	
		Sitosterol	Sitostanol	Sitosterol	Sitostanol	Sitosterol	Sitostanol
Gene name	Gene symbol	10**2 mmol/mol of chol		ug/100 mg chol		ug/100mg chol	
Peroxisome Proliferator-Activated Receptor Alpha	*PPARA*	-0,045	0,192	0,006	-0,040	-0,062	**-,365***
Peroxisome Proliferator-Activated Receptor Gamma (total)	*PPARG_tot*	-0,086	**-,273****	**-,370***	-0,036	-0,270	-0,057
Peroxisome Proliferator-Activated Receptor Gamma (variant 1A)	*PPARGC1A*	0,082	**-,302****	-0,286	-0,155	-0,033	0,086
Peroxisome Proliferator-Activated Receptor Gamma (variant 1B)	*PPARGC1B*	**-,204***	**-,432****	**-,428***	0,109	-0,181	-0,082
Sterol Regulatory Element Binding Transcription Factor 1	*SREBF1_tot*	-0,072	-0,070	-0,253	-0,121	-0,131	0,118
ATP Citrate Lyase	*ACLY*	-0,006	-0,033	-0,202	0,242	-0,054	0,265
Acetyl-CoA Carboxylase Alpha	*ACACA=ACC*	-0,093	**-,271****	-0,300	-0,025	0,045	-0,108
Fatty Acid Synthetase	*FASN*	-0,052	0,004	-0,072	0,247	-0,101	0,269
Fatty Acid Elongase 1	*ELOVL1*	-0,069	0,001	**-,414***	**,496****	-0,125	0,122
Fatty Acid Elongase 2	*ELOVL2*	-0,047	**,331****	0,118	0,321	0,049	0,300
Fatty Acid Elongase 3	*ELOVL3*	0,062	0,098	-0,004	0,128	0,175	0,070
Fatty Acid Elongase 4	*ELOVL4*	-0,125	0,083	-0,118	0,094	-0,066	0,094
Fatty Acid Elongase 5	*ELOVL5*	-0,060	**,306****	0,314	0,074	-0,246	**,352***
Fatty Acid Elongase 6	*ELOVL6*	0,110	0,134	0,185	-0,218	0,303	0,014
Fatty Acid Elongase 7	*ELOVL7*	0,070	-0,043	-0,244	0,152	-0,048	0,015
Stearoyl-CoA Desaturase (Delta-9 Desaturase)	*SCD*	0,040	-0,174	-0,202	0,095	0,007	0,054
Fatty Acid Desaturase 1	*FADS1*	-0,061	0,022	0,109	0,332	-0,291	-0,128
Fatty Acid Desaturase 2	*FADS2*	-0,092	-0,064	-0,018	0,306	-0,207	-0,083
Fatty Acid Desaturase 3	*FADS3*	-0,015	**-,233***	0,011	0,095	-0,234	-0,152
Carnitine Palmitoyltransferase 1A (Liver)	*CPT1A*	-0,046	0,170	-0,161	**,375***	0,146	-0,066
Carnitine Palmitoyltransferase 1B (Muscle)	*CPT1B*	0,170	0,164	-0,201	0,228	0,128	0,156
Glyserol-3 Phosphate Acyltransferase, Mitochondrial	*GPAM=GPAT1*	-0,047	-0,063	-0,303	0,183	-0,031	0,164
Glyserol-3 Phosphate Acyltransferase 2, Mitochondrial	*GPAT2*	0,021	-0,147	-0,272	0,276	-0,082	-0,100
1-Acylglycerol-3-Phosphane O-Acyltransferase 1	*AGPAT1*	**-,270****	0,065	-0,329	0,218	-0,010	0,084
1-Acylglycerol-3-Phosphane O-Acyltransferase 2	*AGPAT2*	-0,065	**-,199***	-0,090	0,059	0,001	0,181
1-Acylglycerol-3-Phosphane O-Acyltransferase 3	*AGPAT3*	0,102	-0,006	0,054	-0,043	0,253	-0,205
1-Acylglycerol-3-Phosphane O-Acyltransferase 4	*AGPAT4*	0,025	-0,007	-0,025	0,141	-0,165	0,058
1-Acylglycerol-3-Phosphane O-Acyltransferase 5	*AGPAT5*	-0,014	-0,077	-0,004	-0,031	-0,210	-0,202
1-Acylglycerol-3-Phosphane O-Acyltransferase 9	*AGPAT9=GPAT3*	0,008	-0,095	-0,034	-0,275	0,153	-0,252
1-Acylglycerol-3-Phosphane O-Acyltransferase 6	*AGPAT6=GPAT4*	-0,014	**-,294****	0,007	-0,321	-0,209	-0,222
Lipin 1	*LPIN1*	-0,082	**-,406****	-0,334	-0,058	0,023	**-,340***
Lipin 2	*LPIN2*	0,155	**-,238***	0,133	**-,444****	0,078	-0,251
Lipin 3	*LPIN3*	-0,001	-0,186	**-,373***	-0,187	-0,138	0,045
Diacylglycerol O-Acyltransferase 1	*DGAT1*	**-,204***	0,047	-0,278	0,174	-0,127	-0,110
Diacylglycerol O-Acyltransferase 2	*DGAT2*	-0,090	0,014	-0,167	0,222	-0,123	0,172
Lipoprotein Lipase	*LPL*	0,000	0,082	-0,019	**,393***	0,179	-0,091
Patatin-Like Phospholipase Domain Containing 2	*PNPLA2=ATGL*	-0,088	-0,010	-0,270	0,048	0,038	-0,111
Lipase, Hormone Sensitive	*LIPE=HSL*	0,056	**,197***	-0,071	-0,013	0,062	-0,169
Perlipin 1	*PLIN1*	-0,005	-0,006	-0,230	0,322	0,028	0,210
Patatin-Like Phospholipase Domain Containing 3	*PNPLA3*	0,026	-0,190	-0,239	0,081	-0,114	-0,020
Phospolipid Phosphatase 2A	*PPAP2A*	0,128	**,318****	0,233	0,118	-0,008	0,104
Phospolipid Phosphatase 2B	*PPAP2B*	-0,135	0,078	-0,167	0,128	0,001	0,290
Phospolipid Phosphatase 2C	*PPAP2C*	0,120	0,139	0,024	0,267	0,135	0,052
Uncoupling Protein 1 (Mitochondrial, Proton Carrier)	*UCP1*	-0,028	0,033	N/A	N/A	-0,137	-0,094
Uncoupling Protein 2 (Mitochondrial, Proton Carrier)	*UCP2*	**-,229***	-0,049	-0,179	**,433***	-0,313	**,343***

Cholesterol metabolism - related genes		Serum n=102		Liver n=38		Bile=41	
		Sitosterol	Sitostanol	Sitosterol	Sitostanol	Sitosterol	Sitostanol
Gene name	Gene symbol	10**2 mmol/mol of chol		ug/100 mg chol		ug/100mg chol	
Nuclear Receptor Subfamily 1, Group H, Member 2	*NR1H2=LXRB*	-0,077	**-,201***	-0,125	0,122	-0,203	0,219
Sterol Regulatory Element Binding Transcription Factor 2	*SREBF2*	-0,105	-0,052	-0,029	**,354***	-0,135	0,205
Acyl-CoA Synthetase Short-Chain Family Member 2	*ACSS2*	-0,013	-0,022	-0,060	0,186	-0,181	-0,122
Acetyl-CoA Acetyltransferase 1	*ACAT1*	-0,048	0,143	-0,050	0,263	0,090	0,228
3-Hydroxy-3-Methylglutaryl-CoA Synthetase 1 (Soluble)	*HMGCS1*	0,046	0,055	0,230	0,179	0,191	0,155
3-Hydroxy-3-Methylglutaryl-CoA Synthetase 3 (Mitochondrial)	*HMGCS2*	-0,084	**,285****	-0,169	-0,016	-0,078	0,180
3-Hydroxy-3-Methylglutaryl-CoA Reductase	*HMGCR*	-0,135	**-,213***	-0,117	0,091	0,037	0,219
Farnesyl-Diphosphate Farnelystransferase 1	*FDFT1*	-0,004	-0,112	-0,018	0,219	-0,018	-0,145
Squalene Epoxidase	*SQLE*	-0,074	-0,100	-0,042	0,308	0,002	0,063
Lanosterol Synthetase (2,3-Oxidosqualene-Lanosterol Cyclase)	*LSS*	-0,019	-0,112	-0,107	0,191	-0,200	**-,324***
Transmembrane 7 Superfamily Member 2	*TM7SF2*	0,003	-0,011	0,068	0,159	-0,067	-0,199
Emopamil Binding Protein (Sterol Isomerase)	*EBP*	0,125	**-,233***	-0,042	-0,134	0,104	**-,331***
Methylsterol Mono-oxygenase 1	*MSMO1=SC4MO*	-0,101	-0,030	0,157	-0,127	-0,046	-0,035
Sterol C5-Desaturase	*SC5DL*	-0,023	**,232***	0,151	0,050	-0,062	0,094
24-Dehydrocholesterol Reductase	*DHCR24*	**-,237***	-0,127	-0,150	-0,110	0,068	0,057
7-Dehydrocholesterol Reductase	*DHCR7*	-0,124	**,224***	-0,083	**,569****	0,122	0,143
ATP-Binding Cassette, Sub-Family A (ABC1), Member 1	*ABCA1*	-0,076	**-,287****	**-,366***	-0,026	-0,031	-0,007
ATP-Binding Cassette, Sub-Family G (WHITE), Member 1	*ABCG1*	0,006	-0,073	-0,207	0,060	0,028	-0,034
ATP-Binding Cassette, Sub-Family G (WHITE), Member 5	*ABCG5*	-0,069	**,243***	-0,073	0,190	-0,138	0,190
ATP-Binding Cassette, Sub-Family G (WHITE), Member 8	*ABCG8*	-0,172	-0,016	-0,310	0,012	-0,209	0,146
Niemann-Pick C1-Like 1	*NPC1L1*	**-,210***	**,248***	-0,168	0,215	-0,295	0,227
Low Density Lipoprotein Receptor	*LDLR*	-0,009	-0,030	0,045	0,005	0,057	-0,244
Proprotein Convertase Subtilisin/Kexin Type 9	*PCSK9*	**-,200***	0,005	-0,186	0,047	-0,038	-0,026
3-Oxoacid CoA Transferase 1	*OXCT1*	-0,011	0,127	-0,249	0,201	0,062	0,301
3-Hydroxybutyrate Dehydrogenase, Type 1	*BDH1*	-0,102	**,229***	-0,119	0,120	0,013	0,026
Scavenger Receptor Class B, Member 1	*SCARB1=SRB1*	-0,128	0,024	-0,139	0,116	-0,158	0,149
Fibroblast Growth Factor 21	*FGF21*	-0,047	-0,135	-0,222	0,111	0,205	**,347***
Cytocrome P450, Family 51, Subfamily A, Polypeptide 1	*CYP51A1*	0,066	**,196***	0,188	0,157	0,032	**-,321***

Supplementary Figure. 1. Serum plant sterols and sitostanol ratios to cholesterol (mean ± SD) in individuals with normal liver (n=44) and NAFLD (n=94). Serum campesterol, sitosterol, avenasterol or sitostanol were not different between normal liver and NAFLD.

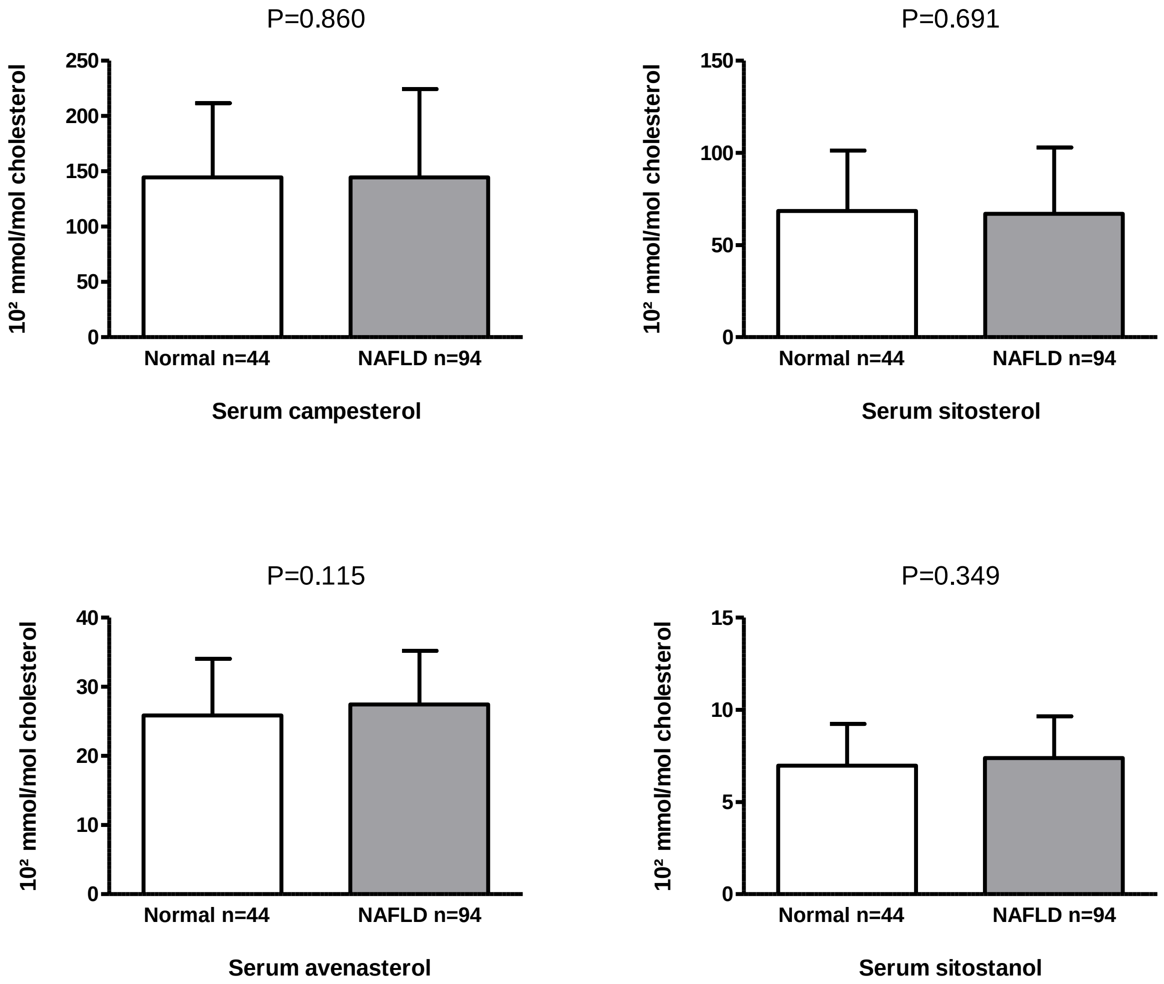

Supplement: Supplementary file 1 [file bsr20171274_Supp1.pdf]
